# Nodding syndrome research, lessons learned from the NSETHIO project

**DOI:** 10.1017/gmh.2019.24

**Published:** 2019-10-18

**Authors:** D. Geelhand de Merxem, J. N. Siewe Fodjo, S. Menon, A. Hotterbeekx, R. Colebunders

**Affiliations:** Global Health Institute, Faculty of Medicine and Health Sciences, University of Antwerp, Antwerp, Belgium

**Keywords:** Epilepsy, etiology, nodding syndrome, onchocerciasis, prevalence

## Abstract

**Background.:**

Until recently, nodding syndrome (NS) was considered as a mysterious disease of unknown etiology. A link between onchocerciasis and epilepsy was suspected for a long time. However, onchocerciasis was not considered as the cause of NS because NS was believed to occur only in onchocerciasis-endemic regions in Uganda, South Sudan, and Tanzania. In October 2015, with funding from the European Research Council, the NSETHIO group launched a trans-disciplinary, multi-country research project to identify the cause of NS and to study the link between onchocerciasis and epilepsy.

**Methods.:**

We reviewed NSETHIO activities as well as all published papers, and compared project findings with results of previous research on NS.

**Results:**

Findings from the NSETHIO project showed that NS is only one of the clinical manifestations in the wide spectrum of onchocerciasis-associated epilepsy (OAE) that could be prevented by strengthening onchocerciasis elimination programs. NSETHIO demonstrated that OAE is an important neglected public health problem in onchocerciasis-endemic areas with no or a sub-optimally functioning onchocerciasis control strategies.

**Conclusions.:**

Today there is overwhelming evidence that NS together with the Nakalanga syndrome is clinical presentations of OAE, a condition that could be prevented by strengthening onchocerciasis elimination programs. While research needs to continue to elucidate the pathophysiological mechanisms causing NS, new strategies to accelerate onchocerciasis elimination coupled with community-based surveillance and treatment programs for epilepsy are urgently needed in areas of high *Onchocerca volvulus* transmission.

## Background

Nodding syndrome (NS) as a distinctive entity was first reported from southern Sudan in the 1990s and investigated by an international team in 2001–2002 (Tumwine *et al*. 2012). The syndrome was initially called nodding disease (Lacey, 2003). However, children with head nodding seizures had been observed already in 1960 in Tanzania (Jilek-Aall, 1964), in 1983 in Liberia (Gerrits, 1983; Van der Waals *et al*. 1983), and in western Uganda (Kipp *et al*. 1994). In 2012, during a conference organized by the Ministry of Health of Uganda, the World Health Organization (WHO) and the Centers for Disease and Prevention in Kampala (Uganda), a consensus was reached to call the condition NS and definitions for suspected, probable, and confirmed cases of NS were proposed (World Health Organization, 2012). At the time of the conference, a link between NS and onchocerciasis was already suspected but the etiology of NS remained obscure.

Today there is overwhelming evidence that NS, together with the Nakalanga syndrome is one of the clinical presentations of onchocerciasis-associated epilepsy (OAE) (Colebunders *et al*. 2018*d*). Surprisingly, it has taken a long time for the scientific community to embrace this evidence. In 1938, Casis already clearly described the association between onchocerciasis and epilepsy (Casis, 1938); in an onchocerciasis-endemic region in Mexico, he reported an epilepsy prevalence of 2.5% (and only a 0.54% prevalence of blind people). Among the persons with epilepsy (PWE), he also described individuals who were mentally impaired, severely stunted, and with delayed development or absence of external signs of sexual development (later described as Nakalanga syndrome (Raper & Ladkin, 1950)). Casis' publication was written in Spanish and was only picked up in 1994 by Jilek-Aall who had equally observed a high number of PWE in Mahenge, an onchocerciasis-endemic region in central Tanzania (Jilek-Aall, 1964). She also suspected a link between onchocerciasis and epilepsy but when she mentioned this at conferences on tropical neurology in Europe in 1994 and 1995, her hypothesis was either met with much scepticism or was rejected outright (Jilek-Aall, 2004). The association between onchocerciasis and epilepsy had also been noticed in Sudan in the 1950s (Kirk *et al*. 1959).

In 2002, Boussinesq described a high prevalence of epilepsy in the Mbam valley, an onchocerciasis-endemic region in Cameroon, and showed that the prevalence of epilepsy was higher in villages located closer to the Mbam river and with higher community microfilarial loads (Boussinesq *et al*. 2002). Moreover, in a case-control study he showed that the frequency and intensity of infection with *Onchocerca volvulus* was higher in people with epilepsy compared to controls (Boussinesq *et al*. 2002).

## Why this reluctance to accept that onchocerciasis is causing nodding syndrome?

1. NS was considered not to occur in other onchocerciasis-endemic areas outside Uganda, South Sudan, and Tanzania and therefore was considered to be related to conditions only present in these countries.
2. NS was considered to be a new epidemic in northern Uganda and South Sudan, potentially induced by factors in relation to the war and insecurity these regions had experienced. However, this could not explain the presence of NS in Mahenge in Tanzania where there had been no war for at least 50 years prior to the detection of NS cases.
3. In recent studies, *O. volvulus* microfilariae (mf) and DNA had not been detected in the cerebrospinal fluid (CSF) of PWE in onchocerciasis-endemic regions (König *et al*. 2010; Tumwine *et al*. 2012; Foltz *et al*. 2013; Colebunders *et al*. 2016*b*). However, prior to the implementation of community-directed treatment with ivermectin (CDTI), several researchers detected *O. volvulus* mf in the CSF of heavily infected persons living in onchocerciasis-endemic areas (Hissette, 1932; Duke *et al*. 1976). It had been suggested that the mf observed in these earlier studies originated from the skin and contaminated the CSF during the procedure. A possible explanation for the absence of mf in CSF in the studies performed during the post-CDTI era could also be the low-mf density of the study participants; in a study in Mahenge, Tanzania, the median mf density was only 1.5 mf/mg (range 0.2–51.5) (König *et al*. 2010).

## The NSETHIO project

In October 2015, RC obtained an advanced European Research Council grant. The title of the grant was ‘Nodding Syndrome: a trans-disciplinary approach to identify the cause and decrease the incidence of river epilepsy’ (https://cordis.europa.eu/project/rcn/198726/factsheet/en); project acronym: ‘NSETHIO.’ The initial NSETHIO hypothesis was based on the fact that there was an epidemiological association between onchocerciasis and epilepsy but the reason for this association could not be explained. Therefore it was suggested that blackflies (*Simulium spp*.), the vector of *O. volvulus*, could transmit either a new neurotropic virus or that the bacteria *Wolbachia*, an endo-symbiont of *O. volvulus,* could cause the epilepsy (Colebunders *et al*. 2014).

An international multi-disciplinary team was established to perform epidemiological, clinical, entomological, and laboratory studies in onchocerciasis-endemic regions in Africa with different degrees of onchocerciasis transmission.

This was the first time that the association between onchocerciasis and epilepsy was investigated in a trans-disciplinary, multi-country, and multi-design study. In doing so, the project collected a large amount of additional evidence that onchocerciasis is able to cause epilepsy. In this paper, we describe the different studies performed by NSETHIO and summarize the most important results. We also discuss the lessons learned from this project and the needs for further research and interventions.

## Methods

We reviewed NSETHIO activities and published papers, and compared project findings with results of previous research on NS.

## Results

Studies performed by the NSETHIO consortium included:

### Prevalence studies

Different door-to-door prevalence studies were performed in onchocerciasis-endemic regions using a two-step methodology. In a first step, homes were visited by village community workers to screen for persons suspected to have epilepsy using a validated 5-item questionnaire. In a second step, persons suspected to have epilepsy were interviewed and examined by a neurologist, trained medical doctor, or clinical officer. Onchocerciasis transmission was assessed by testing children aged 5–10 years for Ov16 antibodies using rapid diagnostic tests (RDT).

A high prevalence of epilepsy was documented in onchocerciasis-endemic regions with a high past or ongoing *O. volvulus* transmission. This was shown in the Bas Uélé (Mukendi *et al*. 2019), Tshopo and Ituri provinces (Levick *et al*. 2017; Lenaerts *et al*. 2018) in the Democratic Republic of Congo (DRC); in the Sanaga and Mbam valleys in Cameroon (Siewe Fodjo *et al*. 2018; Boullé *et al*. 2019); in rural villages in the Mahenge area in Tanzania (Mmbando *et al*. 2018); in northern Uganda (Mbonye *et al*. 2017); and in Maridi county in South Sudan (Colebunders *et al*. 2018*b*). Conversely, in areas of low-onchocerciasis transmission such as the Imo river basin in Nigeria, a low prevalence of epilepsy was observed (Siewe Fodjo *et al*. 2019*d*) (Table 1).

**Table 1.** Summary of epilepsy prevalence studies with Ov16 seroprevalence by the NSETHIO project

Study sites	Population surveyed	Number of PWE (%)	Age of PWE (year): Median (IQR)	Year CDTI started	Ov16 prevalence in children (%)
Mbam Valley, Cameroon (Siewe Fodjo *et al*. 2018)	1321	61 (4.6)	24 (19.5–29.5)	1998	46.9
Sanaga Valley, Cameroon (Siewe Fodjo *et al*. 2018)	204	16 (7.8)	28 (22.25–36.75)	1998	52
Mbam Valley, Cameroon (Boullé *et al*. 2019)	2286	81 (3.5)	24 (20–32)	1998	48.9
Mahenge sub-urban villages, Tanzania (Mmbando *et al*. 2018)	2618	39 (1.5)	26.5 (18.9–36.7)	1997	3.4
Mahenge rural villages, Tanzania (Mmbando *et al*. 2018)	2499	88 (3.5)	25.9 (20.6–35.5)	1997	38.4
Imo river basin, Nigeria (Siewe Fodjo *et al*. 2019*d*)	843	0.5	18 (17.5–24.8)	1994	0
Logo health zone, DRC (Lenaerts *et al*. 2018)	1389	64 (4.6)	17 (13–28)	Never	4.3
Wela, Aketi health zone, (Levick *et al*. 2017; Mukendi *et al*. 2019)	570	39 (6.8)	18 (13.2–23.8)	2003	80.3
Makoko, Aketi health zone, DRC (Levick *et al*. 2017; Mukendi *et al*. 2019)	367	31 (8.4)	18 (13.2–23.8)	2003	46.2

**Fig. 1.**
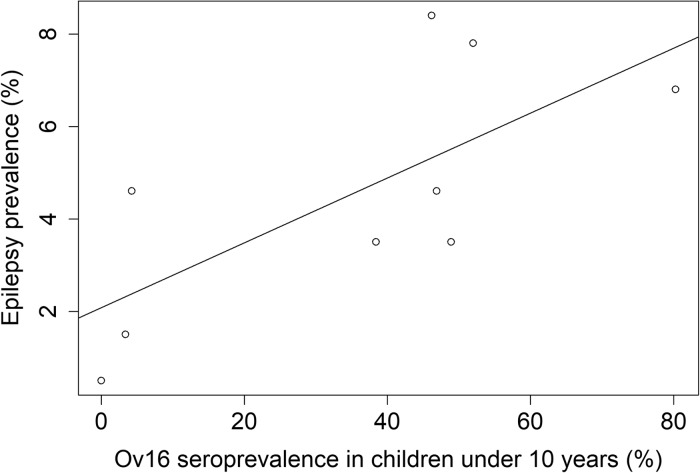
Correlation plot of epilepsy prevalence and Ov16 seropositivity rate.

Upon pooling data from the NSETHIO community-based prevalence studies, a positive correlation was observed between the prevalence of Ov16 antibodies in children aged 5–10 years (as revealed by Ov16 RDT) and the epilepsy prevalence at the study sites; Spearman-*ρ*  =  0.672, *p*  =  0.047 (Fig. 1).

Only in the Logo health zone (DRC), the Ov16 seropositivity rate among children was low despite a high-epilepsy prevalence (Levick *et al*. 2017; Lenaerts *et al*. 2018). It is possible that the children tested in Logo originated from villages where onchocerciasis transmission had been decreasing during recent years as a result of a decreasing number of blackflies because of deforestation. The high-seropositivity rate among older individuals attests to substantial onchocerciasis transmission in the past. However, the high-epilepsy prevalence in Logo could also be partially explained by another cause of epilepsy such as neurocysticercosis.

### Case-control studies

NSETHIO performed four case-control studies in the DRC (Colebunders *et al*. 2016*b*; Levick *et al*. 2017; Mandro *et al*. 2018; Mukendi *et al*. 2019). The lesson learned from these case-control studies is that in order to determine whether onchocerciasis is a risk factor for developing epilepsy, we need to take into account previous ivermectin use as a confounder. This explains why only in the Logo Health zone in Ituri province (DRC), an area where CDTI was never implemented, a clear association between onchocerciasis and epilepsy was observed. PWE in that health zone were two times more likely to present skin mf and had 10 times higher mf densities than age, sex, and village matched controls (Mandro *et al*. 2018). Our studies show that results of case-control studies investigating the association between onchocerciasis and epilepsy performed in areas where CDTI has been implemented, need to be interpreted with great caution.

Two case-control studies in the DRC suggested that ivermectin protected against the development of OAE (Colebunders *et al*. 2016*b*; Levick *et al*. 2017). While the first study included only 18 age-matched pairs, the second study including 96 cases and controls perfectly matched by age, sex, and village (Levick *et al*. 2017). These two cases control studies are very unique because we only assessed the use of ivermectin during the period prior to epilepsy onset in cases, and for the corresponding period in the controls. These studies suggest that *O. volvulus* directly or indirectly causes NS and not a neurotropic virus transmitted by blackflies. Moreover, whole genome sequencing of CSF samples of persons with OAE collected in Titule (DRC) did not reveal any presence of a virus (NSETHIO, unpublished results).

### A cohort study

A cohort study in the Mbam valley in Cameroon showed that the risk to develop epilepsy later in life increased in a dose-response fashion with increasing mf density during childhood (Chesnais *et al*. 2018). Confirmation of these results in a second cohort study would be useful.

### Pre- and post-intervention surveys

Post-CDTI epilepsy surveys were conducted in onchocerciasis-endemic regions where epilepsy prevalence studies were performed prior to the introduction of CDTI or early after the introduction of it. In 2013 in Uganda, no new cases of NS were reported by the Ugandan Ministry of Health after the implementation of biannual treatment with ivermectin coupled with river larviciding (Colebunders *et al*. 2017). This is in contrast to South Sudan, where CDTI has frequently been interrupted and where the NS epidemic is ongoing (Colebunders *et al*. 2016*a*).

In several villages in the Mbam valley in Cameroon, an age shift of PWE was observed from an age ranging between 5–19 to a 20–30 age group after 19 years of CDTI (Siewe Fodjo *et al*. 2018; Boullé *et al*. 2019). This suggests a decreased incidence of OAE in the 5–19 age group. A similar phenomenon was observed in 2017 in northern Uganda (Mbonye *et al*. 2017) (Table 2).

**Table 2.** Age distribution of PWE in pre- and post-intervention studies

Study sites	Age groups	% PWE during baseline survey	% PWE during post-intervention survey	*p* value
Cameroon (Mbam) 1991 *v.* 2017 (Boullé *et al*. 2019)	0–9	9.5	1.2	<0.001
	10–19	58.3	22.2	
	20–29	25.0	42.0	
	⩾30	7.1	34.6	
Cameroon (Bilomo) 1998 *v.* 2017 (Siewe Fodjo *et al*. 2018)	0–9	3.2	11.6	<0.0001
	10–19	60.2	19.7	
	20–29	30.1	47.5	
	30–39	6.5	24.6	
	⩾40	0	6.6	
Uganda (Kitgum and Pader) 2012 *v.* 2017 (Mbonye *et al*. 2017)	0–9	16.7	6.1	<0.0001
	10–19	76.0	55.1	
	20–29	2.1	34.7	
	30–39	2.1	2.0	
	⩾40	3.1	2.0	

### Mathematical modeling

Using mathematical modeling, the NSETHIO consortium estimated that in 2015 between 117 000 and 381 000 people were affected by OAE across onchocerciasis-endemic areas (Vinkeles Melchers *et al*. 2018). Certainly, because of insufficient epilepsy data from onchocerciasis-endemic areas, this is only a very rough estimate. Moreover, the development of OAE is due to high-mf densities and therefore not only onchocerciasis prevalence but also the community microfilarial loads should be taken into account in the model (this was not done). Therefore with the data at hand, it is difficult to arrive at accurate OAE predictions.

### Clinical studies

In all onchocerciasis-endemic areas with high ongoing or recent *O. volvulus* transmission visited by NSETHIO team members, persons with OAE, including nodding seizures and Nakalanga features were identified (Colebunders *et al*. 2018*a*; Siewe Fodjo *et al*. 2019*b*, *c*). It was observed that persons with nodding seizures often have siblings with other forms of epilepsy suggesting that NS and other forms of epilepsy share a common etiological trigger (Colebunders *et al*. 2018*d*). However, a study performed in Maridi in South Sudan showed that persons who develop nodding seizures have more disabilities than persons with other forms of OAE (Colebunders *et al*. 2018*a*). Recently, a correlation between skin mf density and seizure frequency was documented in the DRC (Siewe Fodjo *et al*. 2019*b*).

A simple OAE clinical case definition was proposed (Colebunders *et al*. 2018*d*) and later refined (Colebunders *et al*. 2019). Initially there was a reluctance to use this definition in epidemiological studies (Kaiser *et al*. 2019). However today there is growing support for using this definition as a tool to identify hotspots of high-ongoing onchocerciasis transmission (Mmbando *et al*. 2018; Colebunders *et al*. 2019). In onchocerciasis-endemic regions with high epilepsy prevalence such as in Cameroon and South Sudan, >70% of PWE satisfied the OAE criteria (Colebunders *et al*. 2018*a*; Siewe Fodjo *et al*. 2019*c*).

### Qualitative research

In onchocerciasis-endemic regions with high-ongoing or recent *O. volvulus* transmission, it is common to find households where there are several children with epilepsy, especially in families who reside and/or farm close to blackfly breeding sites (Boussinesq *et al*. 2002; Colebunders *et al*. 2018*b*). The fact that clustering of PWE has been reported among distant, unrelated onchocerciasis-endemic communities makes it unlikely that this is due to a genetic cause. This could rather be because all the children in these families have been exposed to *O. volvulus*-infected blackflies and are more susceptible to develop OAE (Colebunders *et al*. 2018*d*). Because of the clustering of PWE in families, there is a common belief in local communities, even by local health care workers, that epilepsy is contagious and transmissible by contact (Mukendi *et al*. 2019). In Aketi, in the DRC, focus group discussions revealed that children were not accepted in schools because it was considered they could transmit the epilepsy to others. Epilepsy-associated stigma is more likely to occur when there is little knowledge on the correct cause of epilepsy. Therefore information campaigns for health professionals and communities need to be organized to explain why so many children in their villages develop epilepsy. Raising awareness that onchocerciasis can cause epilepsy may reduce stigma and motivate people to take ivermectin to prevent OAE (Siewe & Colebunders, 2019). Another qualitative study in the Logo health zone (DRC) showed that traditional healers played a role in spreading false beliefs about epilepsy, and revealed several barriers that limit access to anti-epileptic treatment, such as limited finances or irregular supply of drugs (Dolo *et al*. 2018).

### Post-mortem study

While the *O. volvulus* infection seems to be able to induce epilepsy, the exact pathophysiological mechanisms underlying OAE are yet to be elucidated (Colebunders & Titulaer, 2017). A recently published post-mortem study of five persons from northern Uganda who died of NS between 2014 and 2017, suggested that NS is a tauopathy and a neurodegenerative disease (Pollanen *et al*. 2018). However, in a post-mortem study performed by the NSETHIO consortium in 2017–2018, among seven persons with NS and two persons with generalized tonic–clonic seizures who died in the same region of northern Uganda, mild to sparse tau-reactive neurofibrillary tangles were observed in four individuals and there was no sign of generalized tauopathy (Hotterbeekx *et al*. 2019). Tau deposits are most likely the consequence and not the cause of NS. Indeed, tau pathology can be induced by seizures themselves (Tai *et al*. 2016) as well as by seizure-associated phenomena, including hypoxia (Raz *et al*. 2018) and repeated head injuries (Santangelo *et al*. 2017).

In addition to field research and publications, the NSETHIO project organized the 1^st^ International workshop on OAE in 2017 (Colebunders *et al*. 2018*c*), during which an OAE alliance was formed to advance OAE research, advocacy, and patient care (https://www.uantwerpen.be/en/research-groups/oae/oae-alliance/).

## Discussion

It can be a daunting task to investigate a condition which is still emerging and poorly understood by the scientific community. Many lessons can be learned from the past 20 years of NS research. These lessons are also useful for other researchers confronted with a relatively new, unexplained phenomenon.

1. All previous reports should be reviewed on the phenomenon, including older reports from the gray literature and those written in other languages than English.
2. A new phenomenon should be investigated in a wider geographical zone than where it was initially discovered. It was a mistake to assume that NS did not occur outside of Uganda, South Sudan, and Tanzania. As a result, past literature from other sites was not considered and field studies were not conducted in other onchocerciasis-endemic regions. If one intends to study the link between onchocerciasis and epilepsy, a good place to start investigating is the DRC, as this is the country in Africa with the highest number of *O. volvulus-*infected persons.
3. Cross-sectional and case-control studies do not fulfill the temporal criterion for causality. Moreover they may fail to consider past ivermectin use as a confounder. Only longitudinal studies like the one conducted in the Mbam valley in Cameroon are able to show a temporal relationship between onchocerciasis and epilepsy.
4. In the past, research teams working on NS focused their research on one or maximum two study sites. The NSETHIO project was able to bring forth new insights because of its multi-country trans-disciplinary (epidemiology, neurology, parasitology, entomology, and modeling) and multi-design approach.
5. When an infectious agent is suspected to be the cause of a disease, it should be reckoned that infectious diseases generally present with a spectrum of clinical manifestations. It is important not only to investigate patients presenting with the end-stage disease, but also to consider that there may be earlier forms of the disease with less pronounced signs and symptoms. Therefore, to determine the spectrum and distribution of the condition, very restrictive and difficult-to-apply case definitions should be avoided. Initially, in order to meet the criteria of a confirmed case of NS, the nodding seizures had to be observed by a trained healthcare worker, or videotaped, or recorded by electroencephalogram (EEG)/electromyogram (EMG) (World Health Organization, 2012); field studies using these criteria in such remote areas is impossible.
6. An absence of reports about a disease in a region does not necessarily imply that it is not prevalent. Indeed, certain diseases or clinical manifestations of diseases may remain unrecognized in remote areas in Africa for many years because of a lack of healthcare and research infrastructure. Furthermore, if a new condition is identified in an area, it is generally only the visible tip of the iceberg.
7. Community-based studies like those conducted by the NSETHIO team necessitate the involvement of village leaders and local healthcare personnel in order to be successful. Indeed, participants were more motivated to participate when sensitized by persons they know. In addition, door-to-door visits were always performed in the presence of a trusted community member so as to ease the contact with the investigated households.
8. OAE research is usually conducted in remote, resource-limited settings and therefore necessitates simple tools that can be used even by local healthcare/community workers. The NSETHIO group showed that community health workers could accurately identify suspected cases of epilepsy in their community, using a validated 5-item questionnaire (Diagana *et al*. 2006). Furthermore, a simple definition for OAE was developed to help estimate the burden of this condition in affected communities (Colebunders *et al*. 2018*d*).
9. Convincing organizations and funders to come on board to address a new, hitherto neglected public health problem, entails challenges which requires that local, national, and regional advocacy capabilities be strengthened. A scientific consortium like the OAE alliance can also constitute a powerful tool to influence decision makers.
10. Investigating the association between onchocerciasis and epilepsy poses major ethical challenges. Firstly, people living in onchocerciasis-endemic areas are very vulnerable because they are poor and mostly illiterate. OAE starts in young children and is often associated with intellectual disabilities. Secondly, epilepsy and onchocerciasis are both stigmatizing conditions. Thirdly, research in an environment with very poor healthcare infrastructure, and little/no access to anti-epileptic treatment is challenging, which raises the question of continuous medical care and treatment for PWE in these settings. Even if an epilepsy treatment program can be set up, its sustainability remains questionable. Whilst in Ituri the NSETHIO project allied itself with the humanitarian organization Malteser and in Bas Uéle with a non-profit organization in Aketi to provide anti-epileptic drugs, the duration of this collaboration is unknown. As a corollary, a long-term uninterrupted anti-epileptic treatment cannot be guaranteed, thereby putting people at risk for life-threatening rebound seizures.

## What is the way forward?

While research needs to continue until the pathophysiological mechanism by which *O. volvulus* is able to trigger epilepsy is elucidated, there is a need to implement and evaluate interventions to prevent and treat epilepsy in onchocerciasis-endemic regions. These should include:

### Prevention of OAE

- Implementing semi-annual CDTI with optimal coverage, with or without ground larviciding of blackfly-infested rivers in a bid to rapidly reduce the incidence of OAE in hyper-endemic settings.
- A clinical trial to investigate whether it is safe to start ivermectin and/or moxidectin before the age of 5 years, because children below that age are already at risk of intense *O. volvulus* infections that predispose to the development of OAE (Chesnais *et al*. 2018).

### Treatment of epilepsy in onchocerciasis-endemic regions

- Comprehensive community-based epilepsy treatment programs, and strategies for surveillance and rapid detection of persons with new onset of epilepsy should be deployed in onchocerciasis hotspots with high-epilepsy prevalence (Siewe Fodjo *et al*. 2019*a*).
- Similar to the morbidity management and disability prevention program (MMDP) developed within Global Programme to Eliminate Lymphatic Filariasis (World Health Organization, 2011), a MMDP for onchocerciasis that incorporates OAE, needs to be developed. Besides providing better care, co-morbidity management has been shown to increase compliance to the mass drug administration programs (Cantey *et al*. 2010). This will not only improve the quality of life of persons with OAE, but will also increase CDTI coverage thereby contributing towards the elimination of the disease by 2025 as projected by the WHO (Dadzie *et al*. 2018).

### OAE advocacy

Long-term advocacy plans are urgently needed to garner the sustained attention of international donors. Alongside public health campaigns to reduce stigma within the community, it is now time that advocacy capacities at local and national levels be further buttressed, which will in turn help advocacy at the regional and global level.

## Conclusions

The NSETHIO project has revealed that in many places in Africa, despite many years of CDTI, the public health problem caused by onchocerciasis is far from being eliminated. Indeed, OAE is a major neglected public health problem in many remote areas with weak onchocerciasis elimination programs. In order to eliminate onchocerciasis and onchocerciasis-associated morbidity and mortality, OAE must be streamlined into onchocerciasis elimination strategies. In epilepsy hotspots, onchocerciasis elimination plans should include sustained campaigns to create awareness about OAE and decrease stigma, alongside enhanced advocacy efforts targeting local, national, regional, and international stakeholders to ensure sustained community-based care programs for affected persons.
